# Intra-Technology Enhancements for Multi-Service Multi-Priority Short-Range V2X Communication

**DOI:** 10.3390/s25082564

**Published:** 2025-04-18

**Authors:** Ihtisham Khalid, Vasilis Maglogiannis, Dries Naudts, Adnan Shahid, Ingrid Moerman

**Affiliations:** IDLab, Department of Information Technology, Imec—Ghent University, Technologiepark Zwijnaarde 126, 9052 Ghent, Belgium; vasilis.maglogiannis@ugent.be (V.M.); dries.naudts@ugent.be (D.N.); adnan.shahid@ugent.be (A.S.); ingrid.moerman@ugent.be (I.M.)

**Keywords:** C-V2X PC5, ITS-G5, CAM, DENM, V2X, latency, enhancement, reliability

## Abstract

Cooperative Intelligent Transportation Systems (C-ITSs) are emerging as transformative technologies, paving the way for safe and fully automated driving solutions. As the demand for autonomous vehicles accelerates, the development of advanced Radio Access Technologies capable of delivering reliable, low-latency vehicular communications has become paramount. Standardized approaches for Vehicular-to-Everything (V2X) communication often fall short in addressing the dynamic and diverse requirements of multi-service, multi-priority systems. Conventional vehicular networks employ static parameters such as Access Category (AC) in IEEE 802.11p-based ITS-G5 and Resource Reservation Interval (RRI) in C-V2X PC5 for prioritizing different V2X services. This static parameter assignment performs unsatisfactorily in dynamic and diverse requirements. To bridge this gap, we propose intelligent Multi-Attribute Decision-Making algorithms for adaptive AC selection in ITS-G5 and RRI adjustment in C-V2X PC5, tailored to the varying priorities of active V2X services. These adaptations are integrated with a priority-aware rate-control mechanism to enhance congestion management. Through extensive simulations conducted using NS3, our proposed strategies demonstrate superior performance compared to standardized methods, achieving improvements in one-way end-to-end latency, Packet Reception Ratio (PRR) and overall communication reliability.

## 1. Introduction

The global connected car market, valued at approximately USD 44.68 billion in 2024, is poised for significant expansion, projected to rise to approximately USD 181.90 billion in 2034. This remarkable growth corresponds to a Compound Annual Growth Rate (CAGR) of 15.1% during the forecast period from 2024 to 2034 [1]. This clearly indicates that vehicular networks are expected to face increased congestion in the future. Moreover, human error is estimated to account for approximately 95% of road traffic accidents in the European Union [2], implying the need for intelligent and autonomous vehicles. The dynamics of V2X networks are highly adaptive, driven by constantly changing network topology and physical environments. To ensure safety and efficiency in such real-time communication, large volumes of data are generated and exchanged between various Cooperative Intelligent Transport Systems (C-ITSs) every second. Depending on the operational environment, V2X communication manifests itself in various forms, including Vehicle-to-Vehicle (V2V), Vehicle-to-Infrastructure (V2I), Vehicle-to-Pedestrian (V2P) and Vehicle-to-Network (V2N) interactions. Cooperative awareness among vehicular nodes will be a cornerstone of the success of next-generation vehicular communication systems. In congested scenarios, advanced vehicular technologies need to prioritize safety for all road users by using state-of-the-art sensing, communication and decision-making capabilities.

In C-ITSs, various message types are used to exchange information between vehicular nodes. Among these, the Cooperative Awareness Message (CAM) is pivotal, providing critical information about a vehicle’s state and behavior, such as its position, speed, heading, acceleration and other relevant data. In addition to CAMs, two other significant message types are Decentralized Environmental Notification Messages (DENMs) and High Priority DENMs (HPDs). DENMs deliver real-time notifications about events that influence traffic safety and efficiency, such as road closures, adverse weather conditions and other noteworthy incidents. HPDs, a specialized subset of DENMs, are reserved for critical and immediate hazards that pose a severe threat to road users, such as emergency braking, serious accidents, or sudden road obstructions. HPDs play a vital role in enabling rapid reactions to avert dangerous situations, such as avoiding a crash site or an unexpected obstacle. All other road safety and traffic-management-related messages in the system with a lower priority than HPDs, DENMs and CAMs are referred to as Low Priority Messages (LPMs). This hierarchical approach to message prioritization ensures that the most critical information reaches its destination reliably without excessive delays, thus safeguarding road users and maintaining traffic flow efficiency.

Two primary short-range V2X technologies exist: ITS-G5 (IEEE 802.11p) and the 3rd Generation Partnership Project (3GPP) Cellular-V2X (C-V2X), which includes LTE-V2X (Release 14) and NR-V2X (Release 16). LTE-V2X Mode 3 (scheduled) relies on the base station for centralized resource allocation, while Mode 4 (autonomous) uses Sensing-Based Semi-Persistent Scheduling (SB-SPS) for distributed selection. NR-V2X follows a similar pattern, with Mode 1 (scheduled) managed by gNB and Mode 2 (autonomous) relying on vehicles. Despite their advantages, both face challenges from spectrum constraints, high packet loads and congestion. This paper focuses on C-V2X PC5 (LTE-V2X Mode 4), but the proposed algorithm is extendable to NR-V2X Mode 2.

The 5.9 GHz ITS safety band supports various V2X services, each assigned a priority to ensure efficient network operation. Table 1 from the European Telecommunications Standards Institute (ETSI) TS 102.636-4-2 [3] defines the prioritization of messages in IEEE 802.11p using Enhanced Distributed Channel Access (EDCA), which categorizes data into Access Categories (ACs) with parameters such as Arbitration Inter-Frame Spacing (AIFS) and Contention Window (CW) sizes to favor high-priority messages and support QoS. However, static AC binding limits adaptability in dynamic V2X environments. ETSI’s Decentralized Congestion Control (DCC) dynamically regulates channel use by adjusting transmission parameters based on real-time load, ensuring reliable, low-latency communication. However, the ETSI reactive DCC framework, as shown in Table 2 [4] relies solely on Channel Busy Ratio (CBR) for rate control, lacking prioritization. Advanced congestion-control strategies are needed to balance channel conditions and message priority for efficient spectrum use in dense V2X networks.

ETSI TS 103 574 [5] defines CR limits for different message priorities in C-V2X PC5, but CAMs and DENMs share the same ProSe Per Packet Priority (PPPP) group [6], which hinders DENM performance in congestion. This grouping assigns similar RRI values to both, limiting the prioritization of critical safety messages. RRI, communicated through Sidelink Control Information messages, controls resource-reservation periodicity, balancing network load and reliability. Although differentiating RRI values could improve performance, its static nature reduces adaptability in dynamic environments. In addition, congestion control can improve radio conditions but can fail at the application layer, risking service reliability. Ensuring that the DCC benefits extend to application layer QoS is crucial for safe and effective V2X communication, particularly in high-density traffic.

To overcome the limitations of current standardized approaches—such as static ACs per message type in ITS-G5 [3], fixed RRIs per message type in C-V2X PC5 [6], the absence of differentiated access between CAMs and DENMs on the PC5 interface [5], and reactive DCC mechanisms that disregard message priority [4]—this paper proposes a set of enhancements for both ITS-G5 and C-V2X PC5 using Multi-Attribute Decision-Making algorithms. The study investigates how intra-technology enhancements for ITS-G5 and C-V2X Mode 4 systems can intelligently adapt resources (ACs, RRIs and transmission rates) to optimize message delivery, particularly for safety-critical applications, in dynamic vehicular environments. Intra-technology enhancement refers to the process of improving the performance, efficiency and reliability of a single short-range V2X communication technology, such as ITS-G5 or C-V2X PC5, by fine-tuning its internal mechanisms, protocols, or parameters. The goal is to maximize the capabilities of the technology within its inherent constraints without involving cross-technology (hybrid) solutions. The key contributions of this paper are as follows:

- Intra-technology enhancement for ITS-G5: Introduces an adaptive AC selection mechanism for ITS-G5, offering a significant advancement in prioritizing different types of C-ITS messages and optimizing the V2X communication performance in terms of latency, Packet Reception Ratio (PRR) and reliability.
- Intra-technology enhancement for C-V2X PC5: Develops an adaptive RRI selection mechanism to enable dynamic, need-based prioritization of C-ITS message types, ensuring efficient resource utilization and timely delivery of critical messages.
- Priority-Aware Rate Control Beyond Reactive DCC: Overcomes the inefficiencies of traditional DCC by introducing a priority-aware variable rate-control mechanism that accounts for CBR, vehicle speed and message priority, thereby achieving a performance-driven approach to congestion control.

The variables used in this study are listed in Table 3. The remainder of the paper is structured as follows: Section 2 reviews related work on the V2X intra-technology enhancements, followed by an overview of standardized approaches for prioritization and congestion control in Section 3. The system model and the problem statement are introduced in Section 4, while Section 5 presents the proposed enhancements for short-range V2X communication technologies. The simulation setup and test scenarios are detailed in Section 6, followed by the results and their analysis in Section 7. Finally, conclusions and directions for future work are provided in Section 8.

## 2. Related Work

### 2.1. ITS-G5: EDCA Access Categories and Congestion Control

A multi-V2X service resource orchestrator for DSRC at the facilities layer is proposed in [7] where a multi-criterion priority function is introduced based on message ranking, usefulness and urgency. The orchestrator disseminates transmission opportunities between V2X services and avoids limitations of the traffic shaping of the access layer. Considering three message types, DENMs, CAMs and CPMs being transmitted in $AC_{VI}$, $AC_{BE}$ and $AC_{BK}$, respectively, a higher performance is observed in terms of DCC access layer queue delay and inter-transmit time. Although useful insights on resource orchestration in a multi-V2X service scenario are shown in [7], intelligent AC allocation among active V2X services is beyond the scope of this work. The performance of ETSI DCC Reactive for ITS-G5 is evaluated in [8] and shortcomings such as channel capacity under-utilization and QoS degradation are shown. To cater for them, an enhanced Reactive DCC approach is presented that is based on channel resource limit instead of transmit rate limit, employing less severe rate control. By sending CAMs and CPMs in $AC_{BE}$ and $AC_{BK}$, respectively, the reception throughput doubles with the proposed adaptations. Again, the performance is limited as higher ACs remain unused in the absence of high-priority messages.

Another work in [9] proposes the dynamic distribution of Basic Safety Messages (BSMs) over the EDCA ACs such that the Packet Error Rate (PER) and the Inter-Packet Gap (IPG) are minimized. For sending ten BSMs per second, the packets are distributed in ratios of 2:3:5, 5:3:2, 6:3:1, 1:3:6, 3:3:4 and 4:3:3 over $AC_{VI}$, $AC_{BE}$ and $AC_{BK}$. Up to 20% reduction in PER and IPG is observed. The use of such an approach in a scenario with multi-V2X services would be limited and can adversely impact the performance of high-priority message types like DENMs and HPDs. The limitation of the ETSI DCC mechanism to account for the priority of the V2X service, the packet size and multiple services per node is criticized in [10]. To address uniform resource allocation, a distributed protocol is proposed in which low-priority message senders allocate resources to high-priority vehicles facing constraints. This involves three steps: calculating resource shortages, notifying other nodes via high-priority message headers and reducing transmission rates of low-priority senders. Although effective in prioritizing, the approach adds overhead due to continuous resource calculations and communication, necessary for adapting to dynamic V2X traffic.

The authors in [11] present an adaptive EDCA mechanism designed for dense WLANs in 5G networks, addressing critical issues of resource-allocation inefficiencies and selfish node behavior in traditional IEEE 802.11 networks. The key contributions include dynamic tuning of Arbitration Inter-Frame Space Numbers (AIFSN) and CW sizes based on active ACs and node density. Additionally, the scheme integrates a game-theoretic approach for transmission probability adaptation, ensuring fairness while penalizing selfish nodes through a novel ‘guidance CW’ concept. Ref. [12] presents a significant advancement in addressing QoS differentiation and fairness in IEEE 802.11-based networks. Unlike traditional methods that rely on static contention parameters (e.g., $CW_{min}$, $CW_{max}$ and AIFSN), Logical EDCA employs logical prioritization to resolve internal contention among ACs deterministically. It standardizes AIFS values across all ACs, simplifying parameter configuration while still maintaining differentiation through its novel internal contention resolution mechanism. The approach balances the needs of high-priority and low-priority traffic, significantly reducing collisions among high-priority ACs and improving the access opportunities for low-priority flows. The performance of Logical EDCA is highly dependent on pre-configured threshold values, which may not adapt optimally to dynamic or unpredictable traffic patterns. The study in [13] introduces dynamic contention window adjustments and AIFSN to optimize the prioritization of safety-critical messages. The proposed method incorporates fairness strategies to ensure equitable access for non-safety messages while prioritizing critical traffic. The study focuses on predefined traffic patterns and does not explore highly dynamic or unpredictable vehicular environments with different message types.

To fulfill application-level QoS constraints for the IEEE 802.11p-based ITS-G5 network in congested scenarios, a study in [14] considered a packet dropping mechanism. It is shown that although rate control improves radio performance, application layer Packet Delivery Rate (PDR) degrades compared to a scenario without a DCC mechanism. A parallel concept was examined by Bazzi et al. [15]. These observations emphasize the importance of further exploration to optimize rate-control procedures for CAMs and other V2X awareness and emergency messages, ensuring that they are transmitted only when meaningful while preserving vehicular situational awareness. The combination of rate control and power control also exists, where a prominent example is the North American Society of Automotive Engineers (SAE) DCC mechanism [16] for DSRC. It derives the rate adaptiveness based on the Linear Integrated Model for Enhanced Rate Control (LIMERIC) [17] and for power adaptations, it employs the Stateful Utilization-based Power Adaptation (SUPRA) framework [18], designed to dynamically manage communication range. A number of studies [19,20,21,22] regarding performance evaluation of SAE DCC have shown notable performance gains over standardized rate-control mechanisms, although with limited improvements from power control. The study in [23] presents a Mixed-Integer Linear Programming-based optimization approach for resilient intersection control of connected and autonomous vehicles under V2X communication. The proposed model enhances traffic efficiency by minimizing travel time and energy consumption while ensuring collision avoidance. Key contributions include a robust trajectory optimization framework that adapts to varying traffic conditions. However, the approach assumes ideal communication reliability, and its computational complexity may hinder scalability in large-scale intersections.

### 2.2. C-V2X PC5: RRI and Congestion Control

A first detailed quantitative evaluation of all existing congestion-control standards for C-V2X and NR-V2X is performed by Brian et al. [24]. Brian et al. also proposed a new RRI DCC approach with three variants based on the ETSI Reactive DCC mechanism, the ETSI Adaptive DCC mechanism and the 3GPP CR limit. The proposed RRI DCC mechanism significantly improves PDR; nonetheless, inter-packet arrival time and neighbor awareness are comparable when benchmarked against standardized approaches. In another study [25], Brian et al. have proposed and comprehensively examined the ETSI adaptive DCC-based $RRI_{Adaptive}$ DCC mechanism. In addition to improving the PDR, $RRI_{Adaptive}$ also overcomes the inherent limitations of table-based mechanisms, particularly addressing the instability of the CBR and the dependence on extensive table tuning.

Among pioneering studies evaluating the impact of DCC on C-V2X PC5 is the work of Mansouri et al. [26]. The performance of the transmission rate reduction based on the 3GPP CR limits (Table 4) is shown to have an adverse impact on the overall congestion of the channel, since multiple vehicles could select the same channel resources. Wendland et al. [27] have introduced a reservation splitting mechanism where a single SB-SPS grant is partitioned into multiple sub-grants with reduced message rate. Under network congestion, individual sub-grants can be selectively disabled while preserving the integrity of the SB-SPS grant mechanism.

Another compelling field-based study by Hu et al. [28] tested congestion by simulating a large deployment of LTE-V2X nodes. This study adapts Modulation and Coding Scheme (MCS) under different congestion conditions and better performance is observed in terms of CBR and PDR. However, the scope of the study is restricted as it focuses on a single packet size, which does not align with the diverse packet size distributions typical of vehicular services, as evidenced by datasets from Renault, Volkswagen [29] and the 3GPP guidelines [30]. In scenarios with varying packet sizes, the effectiveness of MCS adaptation for congestion control depends critically on its ability to minimize subchannel occupation, which is strongly influenced by the underlying packet size distribution [31]. A new scheduling algorithm for LTE-V2X is proposed in [32], named Dynamic Scheduling Algorithm based on Priority Assignment (DSA-PA). It considers traffic classification, SINR of nodes and fairness among safety and non-safety traffic, with the goal of maximizing cell throughput. Specifically for safety traffic, resource blocks are dynamically allocated based on the Average Blocking Rate (ABR) value, which is a measure of the number of safety traffic nodes underserved due to resource block unavailability. Although valid for C-V2X mode 3 with centralized resource allocation, the shown improvements cannot be replicated for C-V2X mode 4 where each node must individually optimize resource selection.

ATOMIC (Adaptive Transmission Power and Message Interval Control), a novel mechanism to enhance the performance of C-V2X Mode 4 in vehicular networks, is proposed in [33]. Critical challenges such as excessive collisions and interference in high-density environments are addressed by jointly optimizing transmission power and message intervals. ATOMIC significantly outperforms standard C-V2X Mode 4 in terms of PDR, particularly in dense vehicular scenarios, achieving an improvement of up to 50% in highly congested environments. Using an NS3-based simulator, the authors in [19] have implemented the DCC algorithm as specified in SAE J2945/1 over the C-V2X access layer. The performance of transmission range and rate is analyzed using metrics such as PDR, IPG and Sidelink Throughput. Based on the analyses, it is revealed that the rate control exerts a more substantial influence on performance compared to the range control.

The study in [34] highlights that the misalignment between message generation and resource allocation in C-V2X PC5 reduces PRR and reliability. Frequent resource allocation, even if unused, helps maintain high PRR and reduces re-selections. An analytical model predicts reselection rates based on generation intervals and allocation periodicity, validated via simulations. Although frequent allocations improve PRR, their impact on resource-limited devices needs further study. In [35], an improved scheduling protocol for C-V2X Mode 4 optimizes CAM transmission by dynamically adjusting the resource re-selection counter RC values, reducing resource waste and inefficiencies. This approach cuts CAM delay by 53.9% and collision probability by 9.52%, though multi-priority scenarios remain unexplored. The study in [36] proposes a multi-agent reinforcement learning-based resource-allocation framework for self-organizing C-V2X communication, optimizing spectrum utilization while minimizing interference. The proposed approach enhances network efficiency, reduces latency and adapts to dynamic vehicular environments, making it suitable for large-scale deployments. However, reliance on reinforcement learning introduces computational overhead, and performance is highly dependent on training quality.

As discussed and summarized in Table 5, most of the literature considers a single message type for performance evaluation or bind message types to specific ACs/RRIs. Also, consideration of realistic multi-service multi-priority scenarios and their respective performance evaluation under different channel loads is missing. In addition, congestion-control schemes usually lack in terms of priority-aware rate control. These considerations, along with the limitations in the standardized approaches [3,4,5,6] constrain the extent of adaptiveness in the selection of intra-technology parameters. To the best of our knowledge, this is the first study in the V2X domain where intra-technology enhancements are considered for jointly managing congestion and traffic prioritization in ITS-G5 and C-V2X PC5 networks.

## 3. Standardized Approaches for Prioritization and Congestion Control in ETSI/3GPP Standards

### 3.1. Traffic Prioritization in ITS-G5 and C-V2X PC5

IEEE 802.11p MAC uses EDCA mechanism to support differentiated QoS by introducing four Access Categories. EDCA is based on DCF, a CSMA/CA algorithm, but adds QoS attributes. Table 1 from ETSI TS 102 636-4-2 [3] shows the mapping of the four ACs ($AC_{VO}$, $AC_{VI}$, $AC_{BE}$ and $AC_{BK}$) to different transmission parameters including intended use. Although this method provides a means to prioritize various message types, its inefficiency stems from the static assignment of each message type to a specific AC, disregarding real-time traffic conditions in the wireless medium. $AC_{VO}$, $AC_{VI}$, $AC_{BE}$ and $AC_{BK}$ are also referred to as AC0, AC1, AC2 and AC3, respectively. This static binding approach serves as a standardized for comparison with the proposed approach later in this paper.

Similarly, for C-V2X PC5, Table 6 from ETSI EN 303 613 V1.1.1 [6] shows the mapping between Traffic Classes (TCs) and PPPP. The table clearly illustrates a static association between message types and their corresponding PPPP values. In particular, the RRI for C-V2X PC5 is typically configured in an ascending manner as the PPPP value decreases. This strategic arrangement ensures more frequent radio resource allocation for high-priority messages, while lower-priority messages are assigned proportionally longer intervals, optimizing resource utilization across varying priority levels. For this study, as a standardized approach, the lower four RRI values 20 ms, 50 ms, 100 ms and 200 ms are statically assigned to HPDs, DENMs, CAMs and LPMs, respectively. Although this standardized approach works fine as far as prioritization is concerned, the lack of real-time adaptiveness in RRI allocation puts limitations on the extent of flexibility and associated performance gains.

### 3.2. Congestion Control in ITS-G5 and C-V2X PC5

The wireless and cellular vehicular communication standards specify the channel conditions that activate congestion-control mechanisms or define the mechanisms themselves to manage network load. DCC techniques can broadly be grouped into three primary categories: Transmission Rate Control (TRC), MCS, Transmission Power Control. These DCC techniques control channel load and improve communication efficiency in dense vehicular environments, balancing the need for reliable communication with effective congestion management.

#### 3.2.1. ETSI ITS-G5 Decentralized Congestion Control (DCC)

ETSI provides the most advanced and comprehensive set of DCC mechanisms for vehicular networks, originally developed for ITS-G5, as outlined in [4]. The primary mechanism used is transmission rate control, which functions by increasing the delay between packet transmissions in response to the CBR. ETSI differentiates between two TRC approaches, DCC Reactive and DCC Adaptive, based on how this delay is calculated. DCC Reactive uses a state machine approach in which each state corresponds to a specific CBR range, as in Table 2. For each CBR level, a defined delay between packets is introduced to regulate transmission rates. This delay, referred to as T_off_, enforces the maximum allowable transmission rate within a given CBR range by setting the time interval before a subsequent packet can be sent. DCC Adaptive, on the other hand, is a rate-control strategy based on the LIMERIC algorithm [17]. Rather than relying on a predefined lookup table, the algorithm dynamically adjusts the packet transmission rate to converge to a target CBR, typically 68%. A detailed analysis of ETSI DCC Adaptive for ITS-G5 can be found in the work by Amador et al. [37].

The reactive DCC mechanism in ITS-G5, which adjusts the transmission rate based on CBR, does not inherently account for message priority. Instead, it applies a uniform adjustment to the transmission rates for all message types (e.g., CAMs, DENMs) based on current channel load without differentiating between their relative priorities. In this system, as congestion increases and the CBR threshold is exceeded, DCC reduces the transmission rates of all messages proportionally, regardless of whether they are high-priority safety messages (such as HPDs, DENMs) or routine status updates (such as CAMs or other LPMs). This approach, while effective in preventing channel overload, may limit the timely delivery of critical information during congestion, as high-priority messages are subject to the same rate reduction as less critical ones. Thus, while reactive DCC provides a straightforward method of controlling channel load, it lacks the granularity to prioritize urgent safety communications, potentially affecting the performance of safety-critical applications under severe congestion.

#### 3.2.2. C-V2X and NR-V2X Congestion Control

In recent years, ETSI has outlined some specific aspects of congestion control for C-V2X and NR-V2X [17]. In particular, they describe methods for measuring the CBR and CR. CBR estimates overall channel congestion by calculating the ratio of subchannels over the last 100 subframes where the sidelink Received Signal Strength Indicator (S-RSSI) exceeds a predetermined threshold. A subchannel represents a set of contiguous or non-contiguous resource blocks within a transmission subframe. CR, in turn, tracks the number of subchannels each vehicle uses over a historical period and includes subchannels reserved for future use based on the current configured grant. As detailed in Table 4, ETSI specifies a maximum CR limit per vehicle, depending on the current CBR measurement. If a vehicle’s CR exceeds this limit for its CBR range, it must reduce the CR by applying a congestion-control mechanism. Although ETSI mentions potential techniques such as packet drop, MCS adaptation, or power control, they do not provide explicit implementation guidelines for these methods. Also, it is interesting to highlight that although PPPP4 and PPPP5 are reserved for DENMs and CAMs respectively in Table 6, they both are part of the same PPPP group in Table 4, which implies no prioritization between them. Using this approach could significantly degrade the performance of DENMs in high-congestion scenarios.

## 4. System Model and Problem Statement

**Figure 1:**
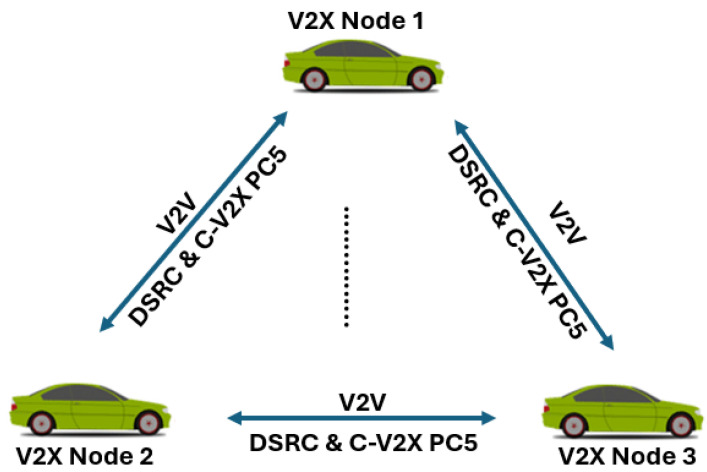
System model for intra-technology enhancements for multi-technology enabled V2X nodes.

The system model for the selection of V2X Intra-technology parameters is described in this section. We consider *N* nodes and for simplicity reasons, only three are illustrated in Figure 1. There is a direct V2V communication link between each pair of V2X nodes. The following assumptions are considered in this regard.

- Each V2X node is equipped with ITS-G5 and C-V2X PC5;
- Each V2X node sends periodic CAMs;
- Each V2X node temporarily sends event-driven messages (HPDs, DENMs) or other LPMs.

For the two short-range technologies, the problem lies in the real-time selection of technology-specific parameters such as the most suitable Access Category and RRI for each C-ITS message type in the system at any particular time. In addition, each node should employ a priority-aware congestion-control mechanism to mitigate the lack of priority consideration in the standardized approaches. This AC/RRI selection and the priority-aware congestion control should maximize the Key Performance Indicators (KPIs), namely latency, PRR and reliability. Let $t_{gen_{X}}^{ITS-G5}$ and $t_{gen_{X}}^{PC5}$ be the generation timestamp added to any C-ITS message *X* transmitted by the V2X node with ITS-G5 and C-V2X PC5, respectively. Similarly, $t_{rec_{X}}^{ITS-G5}$ and $t_{rec_{X}}^{PC5}$ are the current reception timestamp of the V2X node when a C-ITS message *X* is received with ITS-G5 and C-V2X PC5, respectively. The one-way end-to-end (E2E) latency for ITS-G5 ($L_{ITS-G5_{X}}$) and C-V2X PC5 ($L_{PC5_{X}}$) are given as:

(1) $$L_{ITS-G5_{X}}=t_{rec_{X}}^{ITS-G5}-t_{gen_{X}}^{ITS-G5}.$$

(2) $$L_{PC5_{X}}=t_{rec_{X}}^{PC5}-t_{gen_{X}}^{PC5}.$$

where *X* represents any one of the four message types. Let $N_{ITS-G5_{HPD}}$, $N_{ITS-G5_{DENM}}$, $N_{ITS-G5_{CAM}}$ and $N_{ITS-G5_{LPM}}$ be the total number of HPD, DENM, CAM and LPM packets received via ITS-G5, respectively, during a decision window on a V2X node. Similarly, let $N_{PC5_{HPD}}$, $N_{PC5_{DENM}}$, $N_{PC5_{CAM}}$ and $N_{PC5_{LPM}}$ be the total number of HPD, DENM, CAM and LPM packets received via C-V2X PC5, respectively, again, during a decision window on a V2X node. The decision window is a sliding time window during which messages are received and their statistics are observed. Similarly, let $T_{ITS-G5_{HPD}}$, $T_{ITS-G5_{DENM}}$, $T_{ITS-G5_{CAM}}$ and $T_{ITS-G5_{LPM}}$ represent the total number of HPDs, DENMs, CAMs and LPMs sent through ITS-G5, respectively. Similarly, let $T_{PC5_{HPD}}$, $T_{PC5_{DENM}}$, $T_{PC5_{CAM}}$ and $T_{PC5_{LPM}}$ be the total number of HPDs, DENMs, CAMs and LPMs sent through C-V2X PC5, respectively. PRR through ITS-G5 and C-V2X PC5, represented by $PRR_{ITS-G5_{X}}$ and $PRR_{PC5_{X}}$, is defined as:

(3) $$PRR_{ITS-G5_{X}}=\frac{N_{ITS-G5_{X}}}{T_{ITS-G5_{X}}}.$$

(4) $$PRR_{PC5_{X}}=\frac{N_{PC5_{X}}}{T_{PC5_{X}}}.$$

where *X* represents any one of the four message types. Similarly, C-ITS messages are considered reliable if they are received within the latency threshold of each V2X application. The reliability of C-ITS messages for ITS-G5 and C-V2X PC5, corresponding to a specific message type and denoted as $REL_{ITS-G5_{X}}$ and $REL_{PC5_{X}}$, respectively, is defined as:

(5) $$REL_{ITS-G5_{X}}=\frac{N_{ITS-G5_{X}}\;\mathrm{within}\;L_{thr}}{N_{ITS-G5_{X}}}\times 100.$$

(6) $$REL_{PC5_{X}}=\frac{N_{PC5_{X}}\;\mathrm{within}\;L_{thr}}{N_{PC5_{X}}}\times 100.$$

where $L_{thr}$ is the latency performance threshold of the V2X application. This threshold can vary depending on the QoS constraints of the V2X use cases. Rather than using static values for AC in ITS-G5 and RRI in C-V2X PC5 for different message types, this work aims to formulate an intelligent intra-technology parameter-selection algorithm that optimizes latency, PRR and reliability.

## 5. Proposed Enhancements for Short-Range V2X Communication Technologies

The proposed AC adaptation for ITS-G5, RRI adaptation for C-V2X PC5 and priority-aware congestion control for both technologies are described in this section.

### 5.1. ITS-G5 Access Category (AC) Selection

In comparison to the standardized approach where specific ACs are statically linked to specific message types as in Table 1, the proposed algorithm that runs on every V2X node adaptively selects the best AC for each message type based on a number of selection criteria. This includes CBR, the different message types that the node needs to send and the different message types received during the previous sliding time window, denoted as $S_{window}$. For ease of implementation, a fixed $S_{window}$ of 100 ms is used, since it corresponds to the smallest packet generation interval for a message frequency of 10 Hz. $TX_{HPD}$, $TX_{DENM}$ and $TX_{LPM}$ are binary variables that represent whether a particular message type is to be sent (=1) or not (=0). Similarly, $RX_{HPD}$, $RX_{DENM}$, $RX_{LPM}$ are binary variables that represent if messages of a particular message type are received (=1) or not (=0). Including the status of HPD, DENM and LPM receptions in the AC selection decision helps every node to avoid selecting an already in use AC by any other message type in the vicinity. The values of $TX_{CAM}$ and $RX_{CAM}$ remain consistently equal to 1, as periodic CAM transmissions persist continuously within the network. As the simulation always starts with a standardized AC allocation according to Table 1, the proposal is to monitor the CBR and the transmission and reception status of different message types and improve AC allocation, if possible. Since a fixed number of ACs are available, HPDs being the highest priority messages are always assigned to AC0 ($AC_{VO}$). Similarly, LPMs being the lowest priority messages are always assigned to AC3 ($AC_{BK}$).

For CAMs, the approach involves either adhering to the standard $AC_{CAM_{ST}}$ when modifying the AC is not feasible or adjusting the AC (either increasing or decreasing it) when performance improvement is possible. The decision is guided by multiple selection criteria, including the transmission (TX) and reception (RX) statuses of each message type, as well as the CBR. Consequently, the structure of the equation begins with $AC_{CAM_{ST}}$, followed by two additional terms (A and B), as presented in Equation (7).

(7) $$AC_{CAM}=AC_{CAM_{ST}}+A+B$$

Term A, as defined in Equation (8), is formulated as a multi-criteria decision-making process to assess whether assigning a higher AC (the default AC for LPMs) to CAMs could potentially enhance KPIs in specific scenarios. It consists of the product of three components, and the output of term A is binary, giving 0 (indicating the adhesion to the default $AC_{CAM_{ST}}$) or 1 (indicating the reassignment of CAMs to $AC_{LPM}$).

(8) $$A=\left(1-TX_{HPD}\wedge TX_{DENM}\wedge TX_{LPM}\right)\times \left\lceil CBR-CBR_{thr3}\right\rceil \times \left[1-RX_{LPM}\right]$$

The first component, $\left(1-TX_{HPD}\wedge TX_{DENM}\wedge TX_{LPM}\right)$, ensures that not all message types ( $TX_{HPD}$, $TX_{DENM}$ and $TX_{LPM}$) are being transmitted by the node executing the decision-making process, thereby validating the feasibility of AC reassignment. The second component, $\left\lceil CBR-CBR_{thr3}\right\rceil$, determines whether the CBR is high enough to justify an AC change for a meaningful improvement in the KPIs. Among the three CBR thresholds considered, the highest, $CBR_{thr3}$, is used. Finally, the third component, $\left[1-RX_{LPM}\right]$, checks whether the system has detected any LPM receptions within the last sensing window. If LPM receptions are present, the algorithm prevents reassignment of CAMs to the default AC for LPMs, regardless of the CBR or the transmission status of other message types.

Term B, as defined in Equation (9), is also formulated as a multi-criteria decision-making process to evaluate whether assigning a lower AC (the default AC for DENMs) to CAMs could enhance KPIs in specific scenarios. It consists of the product of three components, and its output is binary: either 0 (indicating the adhesion to the default $AC_{CAM_{ST}}$) or −1 (indicating the reassignment of CAMs to $AC_{DENM_{ST}}$).

(9) $$B=\left(1-TX_{HPD}\wedge TX_{DENM}\right)\times \left\lceil CBR_{thr2}-CBR\right\rceil \times \left[\left(RX_{HPD}\vee RX_{DENM}\vee RX_{LPM}\right)-1\right]$$

The first component, $\left(1-TX_{HPD}\wedge TX_{DENM}\right)$, ensures that the V2X node is not simultaneously transmitting both $TX_{HPD}$ and $TX_{DENM}$, thereby validating the feasibility of AC reassignment. The second component, $\left\lceil CBR_{thr2}-CBR\right\rceil$, checks whether the CBR is below a predefined threshold, $CBR_{thr2}$, ensuring that the AC adjustment could significantly improve the KPIs without negatively impacting overall V2X channel utilization. Finally, the third component, $\left[\left(RX_{HPD}\vee RX_{DENM}\vee RX_{LPM}\right)-1\right]$, assesses whether the receptions of HPD, DENM, or LPM were detected during the last sensing window. If any of these message types were received, the system determines whether CAMs can be shifted to $AC_{DENM_{ST}}$.

Similarly, for DENMs, $AC_{DENM}$ is intelligently adapted, as in Equation  (10).

(10) $$\begin{array}{c} AC_{DENM}=AC_{DENM_{ST}}+\left(TX_{HPD}\vee TX_{DENM}\right)\times \left\lceil CBR-CBR_{thr3}\right\rceil \times \left[1-RX_{LPM}\right]+\\ \;\;\;\;\;\;\;\;\;\;\;\;TX_{DENM}\times \left\lceil CBR_{thr2}-CBR\right\rceil \times \left[\left(RX_{HPD}\vee RX_{LPM}\right)-1\right] \end{array}$$

$CBR_{thr1}$, $CBR_{thr2}$ and $CBR_{thr3}$ represent the threshold points between the CBR range from 0 to 1. $AC_{CAM_{ST}}$, $AC_{DENM_{ST}}$, $AC_{HPD_{ST}}$ and $AC_{LPM_{ST}}$ represent the standardized ACs for the CAM, DENM, HPD and LPM packets, respectively. Algorithm 1 highlights the steps involved in the selection of $AC_{CAM}$ and $AC_{DENM}$. SimTime represents the time in milliseconds since the start of the simulation. Performance enhancement with AC adaptation in terms of latency, PRR and reliability is shown in detail in Section 7.
**Algorithm 1** Adaptive Access Category Selection-ITS-G5**Input:** CBR, $TX_{HPD}$, $TX_{DENM}$, $TX_{LPM}$, $RX_{HPD}$, $RX_{DENM}$, $RX_{LPM}$**Output:** Optimal $AC_{CAM}$, $AC_{DENM}$ configuration1:**Initialize**: $AC_{CAM}=AC_{CAM_{ST}}$, $AC_{DENM}=AC_{DENM_{ST}}$, $AC_{HPD}=AC_{HPD_{ST}}$, $AC_{LPM}=AC_{LPM_{ST}}$2:**while** true **do**3:    **if** SimTime % $S_{window}$ = 0 **then**4:        Update CBR value5:        Check current status of $TX_{HPD}$, $TX_{DENM}$ and $TX_{LPM}$6:        Check current status of $RX_{HPD}$, $RX_{DENM}$ and $RX_{LPM}$7:        Calculate $AC_{CAM}$ based on Equation (7)8:        Calculate $AC_{DENM}$ based on Equation (10)9:    **end if**10:**end while**

### 5.2. C-V2X PC5 Resource Reservation Interval (RRI) Selection

The adaptation of RRI in C-V2X PC5 communication significantly enhances resource utilization by introducing flexibility and efficiency in message transmission. By dynamically tailoring RRI values to network conditions, message priorities and application requirements, this approach ensures reliable and timely communication while maintaining optimal resource allocation. In contrast, the static methodology described in Table 6 from ETSI EN 303 613 V1.1.1 [6] requires a fixed PPPP value for each message type, leading to static RRI allocations and inherent performance limitations, as elaborated in Section 7.

The proposed adaptive algorithm enables each node to select RRI values dynamically based on multiple input parameters, such as CBR, the types of messages transmitted ($TX_{HPD}$, $TX_{DENM}$) and the types of messages received ($RX_{HPD}$, $RX_{DENM}$). These transmission and reception parameters, as previously discussed in the AC adaptation subsection, provide a comprehensive view of the messaging environment. V2X nodes use this information to adapt their RRI, aiming to optimize KPIs in a sliding time window of 100 ms ($S_{window}$). A generalized closed-form expression for the selection of RRI is presented in Equation (11), which provides flexibility to incorporate customizable values for the CBR thresholds ($CBR_{thr1}$, $CBR_{thr2}$ and $CBR_{thr3}$). Moreover, the inclusion of RX parameters ($RX_{HPD}$, $RX_{DENM}$) is crucial as it offers a holistic perspective on the types of messages being exchanged within the node’s vicinity. Importantly, any RRI adaptation mechanism must ensure performance improvements for one node without compromising the performance of others. Equation (11) defines the RRI for the highest priority message type present in the system, such as HPD, DENM, or CAM. Lower-priority messages dynamically adapt to higher RRI values in a hierarchical sequence. For example, in the absence of HPDs, DENMs inherit the RRI value derived from Equation (11). Suppose that this calculated RRI is 20 ms; in such a configuration, the ubiquitously present CAMs would be assigned an RRI of 50 ms, while LPMs, if active, would adopt an RRI of 100 ms. This priority-based cascading mechanism ensures a systematic allocation of RRI values across message types without violating priority ranking among them.

(11) $$RRI=TX_{HPD}+TX_{DENM}*V1*TX_{HPD}^{\prime }+V2*TX_{HPD}^{\prime }*TX_{DENM}^{\prime }$$

where the components V1 and V2 are defined as follows:

(12) $$\begin{array}{c} V1=RX_{H}*(\left\lceil CBR_{thr2}-CBR\right\rceil +\left(\left\lceil CBR-CBR_{thr2}\right\rceil -\left\lceil CBR-CBR_{thr3}\right\rceil \right)*2+\\ \;\;\left\lceil CBR-CBR_{thr3}\right\rceil *3)+RX_{DENM}*\left(\left\lceil CBR_{thr3}-CBR\right\rceil +\left\lceil CBR-CBR_{thr3}\right\rceil *2\right)+\\ \;\;\;\;\;\;\;\;\;\;\;\;\;\;\;\;\;\;\;\;\;\;\;\;\;\;RX_{HPD}^{\prime }RX_{DENM}^{\prime } \end{array}$$

(13) $$\begin{array}{c} V2=RX_{HPD}*(\left\lceil CBR_{thr1}-CBR\right\rceil +\left(\left\lceil CBR-CBR_{thr1}\right\rceil -\left\lceil CBR-CBR_{thr2}\right\rceil \right)*2+\\ \;\;\;\left\lceil CBR-CBR_{thr2}\right\rceil *3)+RX_{DENM}*(\left\lceil CBR_{thr2}-CBR\right\rceil +(\left\lceil CBR-CBR_{thr2}\right\rceil -\\ \;\;\;\;\;\;\;\;\;\left\lceil CBR-CBR_{thr3}\right\rceil )*2+\left\lceil CBR-CBR_{thr3}\right\rceil *3)+RX_{HPD}^{\prime }RX_{DENM}^{\prime } \end{array}$$

Algorithm 2 outlines the procedural steps for selecting the optimal RRI. For example, if a node is exclusively transmitting CAMs, it may opt for shorter RRI values in scenarios devoid of DENMs or HPDs. However, the node simultaneously monitors the received messages to assess whether any neighboring node is transmitting higher-priority messages (HPDs or DENMs). If higher priority messages are detected, the node switches to the default RRI allocation to avoid performance degradation of high-priority messages. In contrast, in the absence of such messages, the node can confidently utilize lower RRI values for CAM transmission, leveraging the temporary network state while ensuring minimal impact on overall network congestion. This adaptation process continuously considers the overarching CBR, striking a balance between efficient selection of RRI and the preservation of the performance integrity of high priority messages.

This thoughtful and context-aware approach aims to optimize RRI selection dynamically for every message type on each node, preventing congestion and maintaining performance for critical messages. In doing so, it improves the overall communication efficiency of the network without introducing constraints or risks to the delivery of high-priority messages.
**Algorithm 2** Adaptive RRI Selection - C-V2X PC5**Input:** 
CBR, $TX_{HPD}$, $TX_{DENM}$, $RX_{HPD}$, $RX_{DENM}$**Output:** 
Optimal $RRI_{HPD}$, $RRI_{DENM}$, $RRI_{CAM}$, $RRI_{LPM}$ configuration1:**Initialize**: $RRI_{HPD}=20$, $RRI_{DENM}=50$, $RRI_{CAM}=100$, $RRI_{LPM}=200$2:**while** true **do**3:    **if** SimTime % $S_{window}$ = 0 **then**4:        Update CBR value5:        Check current status of $TX_{HPD}$, $TX_{DENM}$6:        Check current status of $RX_{HPD}$, $RX_{DENM}$7:        Calculate RRI based on Equation (11)8:        Sequentially assign RRIs to applicable message types9:    **end if**10:**end while**

### 5.3. Priority-Aware Rate Adaptation

Building on the limitations of the Reactive DCC mechanism of ETSI [4], as discussed in Section 3.2, this section introduces an intelligent and priority-aware rate adaptation algorithm. Unlike the standardized approach in Table 2, which mitigates congestion by uniformly reducing rates across all message types, the proposed Algorithm 3 adopts a more nuanced strategy. This method considers not only the CBR but also the Speed (between 0 and 1, a Min–Max normalized value for the speed values in km/h from 0 to 120 km/h) of the nodes, allowing for a more dynamic and context-sensitive adaptation. The algorithm prioritizes the message types according to their importance. HPDs are transmitted at the maximum rate (10 Hz in this study), regardless of CBR or Speed, ensuring their critical delivery. In contrast, other messages such as DENMs, CAMs and LPMs undergo differentiated rate control: DENMs experience the least stringent reduction under congestion, whereas LPMs are subject to the most stringent rate control. Generalized closed-form expressions for message frequencies ($F_{DENM}$, $F_{CAM}$ and $F_{LPM}$) are provided in Equations (14)–(16), offering flexibility through customizable CBR thresholds ($CBR_{thr1}$, $CBR_{thr2}$ and $CBR_{thr3}$). $f_{max}$ and $f_{min}$ are parameters specific to V2X services that define the maximum and minimum message transmission rates for each message type. The minimum rate, $f_{min}$, is fixed at 1 Hz for all V2X services. In contrast, $f_{max}$ is set to 10 Hz for CAMs, DENMs and HPDs, while it is significantly higher at 200 Hz for LPMs via ITS-G5 and 500 Hz for LPMs via C-V2X PC5. This elevated $f_{max}$ for LPMs is deliberately chosen to create congestion scenarios, allowing a thorough evaluation of both short-range V2X communication technologies. Furthermore, the inclusion of the Speed parameter ensures that the rate control dynamically adapts to abrupt traffic changes, mitigating potential PRR degradation and improving the robustness of the system.

(14) $$\begin{array}{c} F_{DENM}=\left\lceil CBR_{thr2}-CBR\right\rceil *f_{max}+\left(\left\lceil CBR-CBR_{thr2}\right\rceil -\left\lceil CBR-CBR_{thr3}\right\rceil \right)*\\ \;\;\;\;\;\;\;\;\;\;\;\;\;\left(6+\left\lceil 4*Speed\right\rceil \right)+\left\lceil CBR-CBR_{thr3}\right\rceil *\frac{f_{max}}{2} \end{array}$$

(15) $$F_{CAM}=\left\lceil CBR_{thr1}-CBR\right\rceil *f_{max}+\left\lceil CBR-CBR_{thr1}\right\rceil *\left(\left(3\left\lceil 4*CBR\right\rceil -2\right)-\left\lceil 4*Speed\right\rceil \right)$$

(16) $$\begin{array}{c} F_{LPM}=\left\lceil CBR_{thr1}-CBR\right\rceil *f_{max}+\left(\left\lceil CBR-CBR_{thr1}\right\rceil -\left\lceil CBR-CBR_{thr2}\right\rceil \right)*\\ \;\;\;\;\;\;\;\;\;\;\;\;\;\;\;\;\;\;\;\frac{f_{max}}{2}+\left\lceil CBR-CBR_{thr2}\right\rceil *f_{min} \end{array}$$

**Algorithm 3** Priority-Aware Rate Adaptation—ITS-G5 and C-V2X PC5**Input:** 
CBR, Speed**Output:** 
Optimal $F_{DENM}$, $F_{CAM}$ and $F_{LPM}$ selection1:**Initialize**: $F_{HPD}$ = 10, $F_{DENM}$ = 10, $F_{CAM}$ = 10 and $F_{LPM}$ = 200 (ITS-G5)/500 (C-V2X PC5)2:**while** true **do**3:    **if** SimTime % $S_{window}$ = 0 **then**4:        Update CBR, Speed5:        Calculate $F_{DENM}$ based on Equation (14)6:        Calculate $F_{CAM}$ based on Equation (15)7:        Calculate $F_{LPM}$ based on Equation (16)8:    **end if**9:**end while**

## 6. Simulation Setup and Description of Scenarios

### 6.1. Simulation Setup

To evaluate the proposed enhancements for AC/RRI adaptation and congestion control, we use ms-van3t [38], an NS3-based multi-stack ETSI-compliant V2X framework. ms-van3t provides NS3 modules to build and simulate ETSI-compliant V2X applications using SUMO (v-1.6.0+). ms-van3t provides both ITS-G5 and C-V2X PC5 capabilities. The simulation consists of a 3000 m straight road in SUMO with a total of ten V2X nodes, where each node is within the proximity of the others. The nodes enter the simulation with variable delay. Every node is equipped with both short-range technologies; however, they operate at different frequencies, and there is no interference between them. The results are averaged over 10 iterations with random seeds and repeated for a low-speed scenario (vehicle speed capped at 35 km/h) and a high-speed scenario (vehicle speed capped at 120 km/h). The simulation parameters specific to ITS-G5 and C-V2X PC5 are summarized in Table 7.

### 6.2. Description of Scenarios

To rigorously validate the proposed adaptive algorithms, four representative scenarios were meticulously designed, each simulating realistic vehicular communication environments. In all scenarios, CAMs are consistently transmitted, while HPDs, DENMs and LPMs are selectively included to reflect need-based behavior. These scenarios serve a dual purpose: (1) to emulate real-world conditions and (2) to evaluate algorithms under diverse network parameters, including varying CBR, node Speed and the mix of transmitted and received message types. The scenarios were uniformly implemented and evaluated with the ITS-G5 and C-V2X PC5 technologies to ensure a fair comparative analysis. A detailed breakdown of the scenarios is as follows:

- Scenario 1 (Low-Congestion): All nodes transmit only CAMs, resulting in minimal network congestion. This scenario provides a benchmark for performance metrics such as one-way end-to-end latency, PRR and reliability in an ideal, low-contention environment.
- Scenario 2 (Moderate-Congestion with LPMs): All nodes send periodic CAMs alongside high-intensity LPM messages, creating a significantly congested environment. This setup evaluates the adaptive algorithm’s performance under moderate-to-high network contention, focusing on scenarios where LPMs dominate alongside CAMs.
- Scenario 3 (High-Congestion with DENMs and LPMs): Along with CAMs and LPMs, 20% of the nodes also transmit DENMs. This scenario simulates a congested network where high-priority messages (DENMs) must compete for resources in the presence of CAMs and LPMs, providing critical insights into the adaptive algorithm’s ability to prioritize high-importance messages effectively.
- Scenario 4 (Extreme-Congestion with All Message Types): All nodes transmit CAMs and LPMs, while 20% of the nodes also transmit DENMs and 20% transmit HPDs. This scenario represents a worst-case scenario with all four message types in operation, utilizing all ITS-G5 ACs and C-V2X PC5 RRI levels. Although rare in real-world applications, this stress test is vital to ensuring that the proposed algorithms maintain robustness and efficiency under extreme contention conditions.

These scenarios are summarized in Table 8. By covering a wide spectrum of network conditions, these scenarios provide a comprehensive evaluation of the proposed enhancements, highlighting their adaptability and effectiveness across varying levels of congestion and message-prioritization challenges.

## 7. Results and Analysis

This section presents the results for ITS-G5 and C-V2X PC5 for the four scenarios outlined in Section 6. For each scenario, simulations were performed for both the adaptive approach (denoted as ’Proposed’ in the figures) and the standardized case (denoted as ’Standard’ in the figures) to demonstrate improvements in KPIs. The results are averaged over ten iterations with different random seeds to improve robustness and reduce variance.

### 7.1. ITS-G5

**Figure 2:**
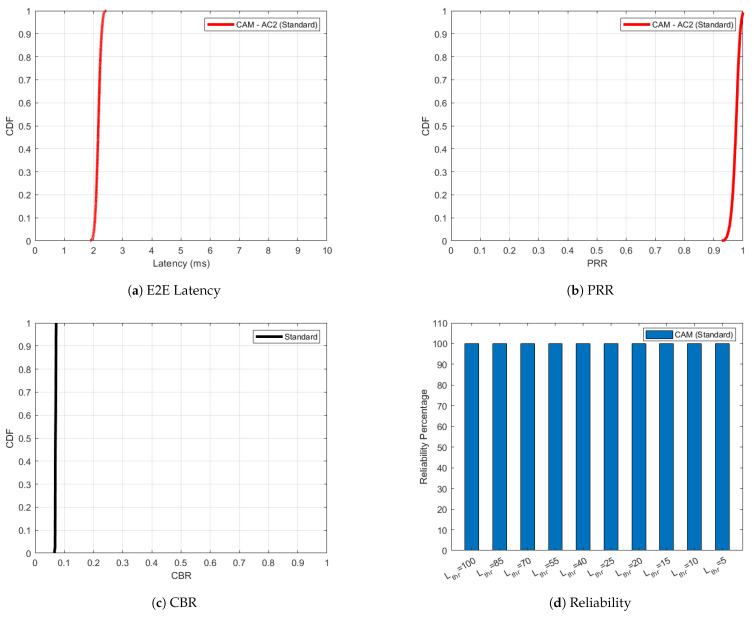
Optimal performance for ITS-G5 ‘Standard’ case in scenario 1 with respect to one-way end-to-end latency, PRR, CBR and reliability, negating the need for further improvement through the proposed approach.

**Figure 3:**
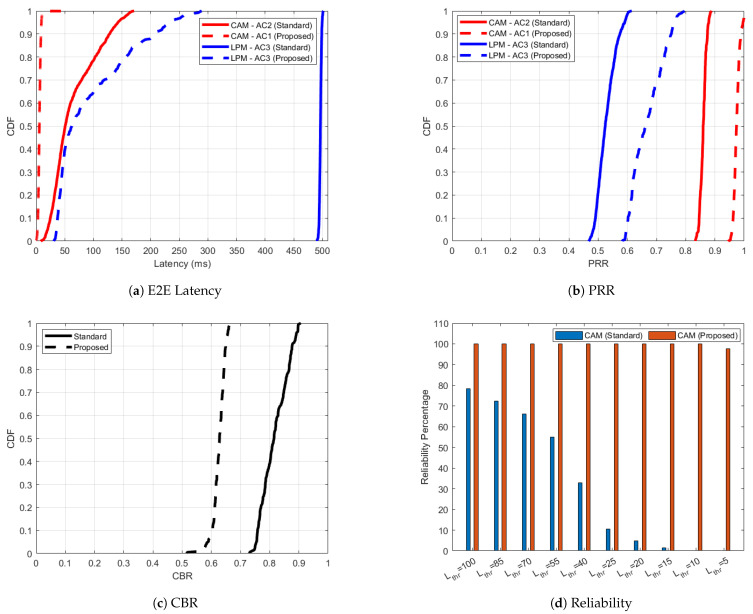
ITS-G5 performance improvement for the ‘Proposed’ versus ‘Standard’ cases in scenario 2 with respect to one-way end-to-end latency, PRR, CBR and reliability.

**Figure 4:**
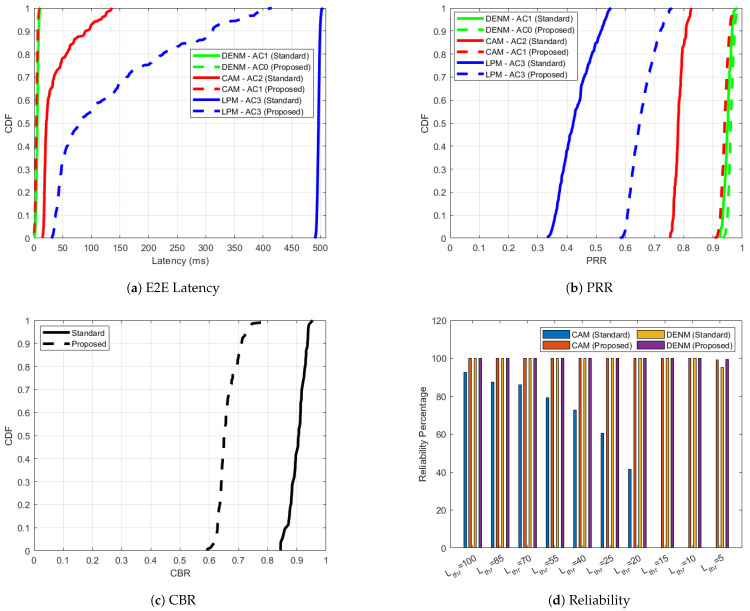
ITS-G5 performance improvement for the ’Proposed’ versus ’Standard’ cases in scenario 3 with respect to one-way end-to-end latency, PRR, CBR and reliability.

**Figure 5:**
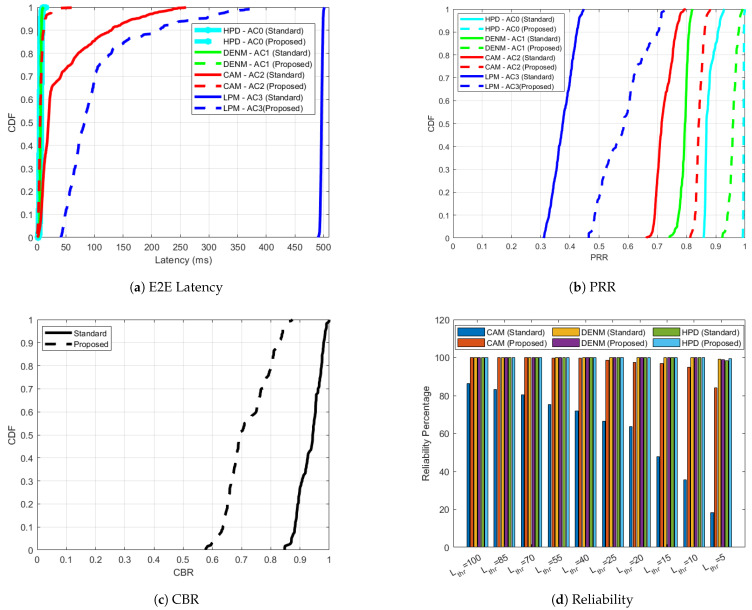
ITS-G5 performance improvement for the ’Proposed’ versus ’Standard’ cases in scenario 4 with respect to one-way end-to-end latency, PRR, CBR and reliability.

The simulation results for ITS-G5 under low speed conditions, with a maximum speed restricted to 35 km/h, are presented in Figure 2, Figure 3, Figure 4 and Figure 5 for the four scenarios evaluated. Figure 2 illustrates the performance of the standardized approach in scenario 1, where CAMs are transmitted using AC2 ($AC_{BE}$), as defined in Table 1. As evident in Figure 2c, the network experiences minimal congestion between 7–8% with each node in the simulation only transmitting CAMs at a frequency $F_{CAM}$ of 10 Hz. This low congestion translates into exceptional performance metrics: the one-way end-to-end latency remains minimal in Figure 2a, while the PRR in Figure 2b approaches 100%. Taking into account a number of latency thresholds, $L_{thr}$, ranging from moderate (100 ms) to very strict (5 ms), the reliability of the CAM in Figure 2d is always 100%. Given the optimal conditions in this scenario, adaptiveness is unnecessary as no additional performance enhancements are required.

In scenario 2 of ITS-G5 (Figure 3), the introduction of high intensity LPMs significantly increases bandwidth consumption, as evidenced by the increase in CBR in Figure 3c. This elevated contention adversely affects the network, leading to a noticeable increase in CAM latency (Figure 3a) when CAMs are statically assigned to AC2. This increased congestion leads to higher chances of collisions leading to packet losses. $PRR_{ITS-G5_{CAM}}$ of 0.99 for the standard case (’CAM-AC2 (Standard)’) in scenario 1 is reduced to 0.87 for the standard case (‘CAM-AC2 (Standard)’) in scenario 2. LPMs also experience severe latency, ranging between 450 and 500 ms. These high contention levels result in a degradation of PRR for both CAMs and LPMs, as shown in Figure 3b. To address these challenges, the proposed AC adaptation algorithm dynamically reallocates CAMs from AC2 to AC1 in the absence of HPDs and DENMs, while LPMs, as low-priority messages, remain on AC3. The proposed AC adaptation algorithm detects vacant ACs originally reserved for DENMs/HPDs and utilizes them for sending CAMs, not sticking to the default AC for CAMs. With this approach, even in congested scenarios, CAMs succeed in obtaining more transmission opportunities compared to the standard case. CAMs will contend less with lower-priority messages (like LPMs in our study), leading to fewer delays and reduced risk of being dropped in congested scenarios, hence improvement in the PRR. During the same time, the intelligent rate-control algorithm is activated, reducing contention and optimizing network performance. This observation is evidenced by the decrease in CBR, as demonstrated by the CBR curve corresponding to the ’Proposed’ scenario in Figure 3c. With the proposed enhancements, the 95percentile of $PRR_{ITS-G5_{CAM}}$ improves significantly to 0.99 compared to 0.87 in the standardized scenario. Due to priority-aware congestion control, the 95 percentile of $PRR_{ITS-G5_{LPM}}$ increases to 0.76 (‘LPM-AC3 (Proposed)’) from 0.59 (‘LPM-AC3 (Standard)’). The 95 percentile of CAM latency ($L_{ITS-G5_{CAM}}$) is reduced from 142.81 ms in the standardized case ‘CAM-AC2 (Standard)’ to just 4.14 ms in the adaptive case ‘CAM-AC1 (Proposed)’. Figure 3d further illustrates the reliability of CAM transmissions under different latency thresholds. With AC adaptation, CAMs are 99% reliable even for strict $L_{thr}$ of 10 ms, whereas, for the similar threshold, none of the CAMs received in ’CAM (Standard)’ case are reliable. This demonstrates the effectiveness of the proposed adaptive mechanism in mitigating high contention and optimizing network performance.

In ITS-G5 scenario 3 (Figure 4), 20% of the nodes transmit DENMs in addition to the already congested channel carrying CAMs and LPMs. This increased network load shifts the CBR curve for the ’Standard’ case further to the right in Figure 4c, reflecting increased contention. This scenario is particularly significant because DENMs carry critical safety information, making it imperative to optimize KPIs through adaptive mechanisms. Similarly to scenario 2, the adaptive approach reallocates CAMs from AC2 to AC1 and promotes DENMs from AC1 to AC0 in the absence of HPDs. This strategic update of $AC_{CAM}$ and $AC_{DENM}$, combined with the intelligent priority-aware rate-control algorithm, reduces overall channel congestion. The resulting improvement is evident in the CBR curve, with its 95 percentile value reduced to 0.73 for the ’Proposed’ case, compared to 0.94 for the ’Standard’ case, as shown in Figure 4c. The adaptive enhancements also improve the latency metrics. The 95 percentile of DENM latency ($L_{ITS-G5_{DENM}}$) decreases from 4.99 ms in the standardized case ’DENM-AC1 (Standard)’ to 3.5 ms in the adaptive case ’DENM-AC0 (Proposed)’, as shown in Figure 4a. Similarly, in Figure 4b, the PRRs see significant improvements: the 95 percentile of $PRR_{ITS-G5_{DENM}}$ increases from 0.96 to 0.98, $PRR_{ITS-G5_{CAM}}$ increases from 0.81 to 0.96 and $PRR_{ITS-G5_{LPM}}$ jumps from 0.53 to 0.73. The reliability of DENMs is particularly robust, even under stringent latency thresholds, as shown in Figure 4d. While both ’DENM (Standard)’ and ’DENM (Proposed)’ cases exhibit high reliability at a 5 ms latency threshold, the adaptive approach ’DENM (Proposed)’ demonstrates slightly better reliability. In particular, the proposed adaptations produce substantial improvements in CAM reliability compared to the standardized case, enhancing the effectiveness of the algorithm in high-congestion scenarios involving critical message types.

Scenario 4 represents the most constrained and challenging case for ITS-G5, with a highly saturated network environment. In this scenario, 60% of the nodes transmit only CAMs and LPMs, 20% transmit DENMs along with CAMs and LPMs and the remaining 20% send HPDs along with CAMs and LPMs. Unlike earlier scenarios, AC adaptation is no longer feasible due to the simultaneous presence of all four message types in the V2X network. Consequently, the nodes rely solely on the proposed priority-aware rate-control algorithm to improve KPIs, as depicted in Figure 5. In the standardized case without rate control, the network exhibits suboptimal performance for critical messages: the 95 percentile of $PRR_{ITS-G5_{HPD}}$ and $PRR_{ITS-G5_{DENM}}$ are 0.91 and 0.81, respectively, well below the ideal reliability levels required for high-priority communication. The CBR curve for the ’Standard’ case, shown in Figure 5c, is significantly shifted to the right compared to scenarios 2 and 3, indicating severe channel congestion. By employing the proposed priority-aware rate adaptation algorithm, the overall congestion is reduced, as evidenced by the 95 percentile CBR value of 0.84 in the ’Proposed’ case compared to 0.98 in the ’Standard’ case. The improvements in latency are substantial, as shown in Figure 5a: the 95 percentile latency for HPDs ($L_{ITS-G5_{HPD}}$) decreases from 4.12 to 3.31 ms, for DENMs ($L_{ITS-G5_{DENM}}$) from 3.94 to 3.55 ms and for CAMs ($L_{ITS-G5_{CAM}}$) from 168.6 to 10.35 ms. In terms of PRR (Figure 5b), $PRR_{ITS-G5_{HPD}}$ improves from 0.91 to 0.99, $PRR_{ITS-G5_{DENM}}$ from 0.81 to 0.98, $PRR_{ITS-G5_{CAM}}$ from 0.77 to 0.87 and $PRR_{ITS-G5_{LPM}}$ from 0.43 to 0.71. HPDs and DENMs, using AC0 and AC1, respectively, maintain exceptional reliability levels ranging from 98% to 100% across all latency thresholds, as shown in Figure 5d. Notably, ’CAM (Proposed)’ demonstrate over 95% reliability even under a stringent latency threshold ($L_{thr}$) of 10 ms. In contrast, ’CAM (Standard)’ achieves a significantly lower reliability of only 35% under the same conditions.

To improve readability and facilitate comprehensive analysis, Table 9 and Table 10 presents the 95 percentile values of latency, PRR and CBR for both standardized and adaptive cases across all scenarios in ITS-G5 at a low speed of 35 km/h and a high speed of 120 km/h, respectively. The results highlight that congestion levels diminish at elevated speeds due to the increased inter-vehicle distances maintained for safety purposes. However, this improvement is accompanied by a reduction in PRR, primarily due to the fixed transmission power configuration. Although theoretically higher safety distances at elevated speeds should lead to increased latency, actual observed latency values are also influenced by the CBR and the waiting time for channel access.

### 7.2. C-V2X PC5

**Figure 6:**
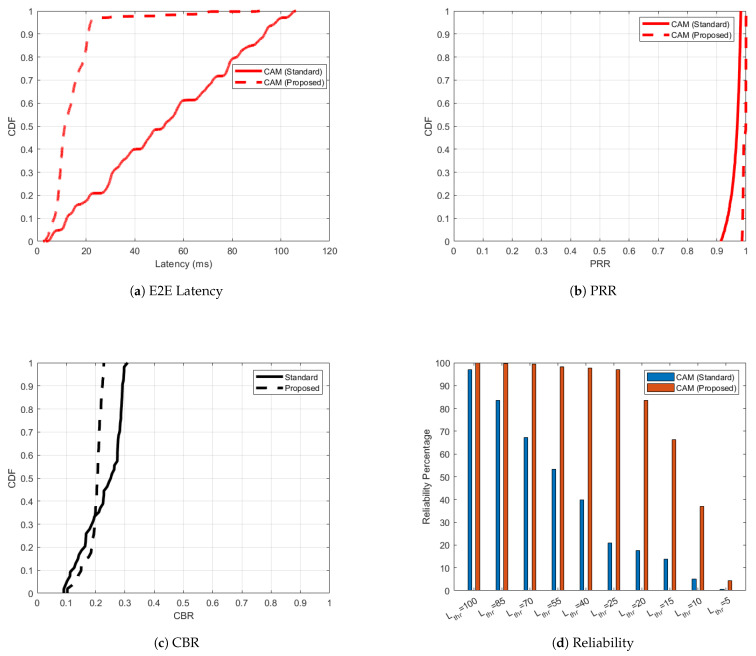
C-V2X PC5 performance improvement for the ’Proposed’ versus ’Standard’ cases in scenario 1 with respect to one-way end-to-end latency, PRR, CBR and reliability.

**Figure 7:**
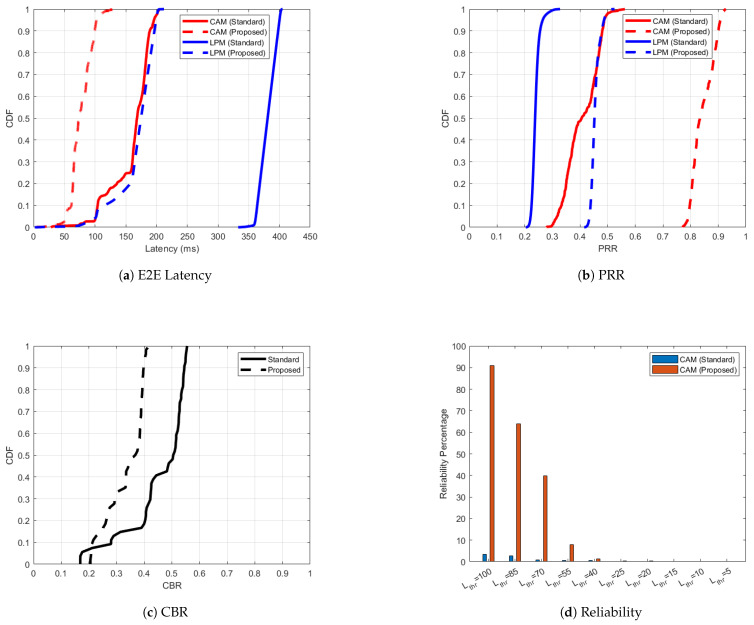
C-V2X PC5 performance improvement for the ‘Proposed’ versus ‘Standard’ cases in scenario 2 with respect to one-way end-to-end latency, PRR, CBR and reliability.

**Figure 8:**
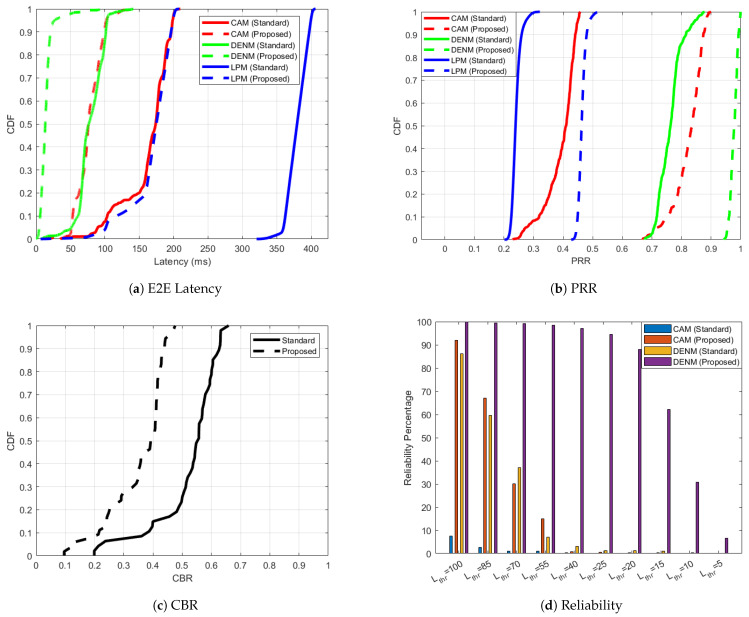
C-V2X PC5 performance improvement for the ‘Proposed’ versus ’Standard’ cases in scenario 3 with respect to one-way end-to-end latency, PRR, CBR and reliability.

**Figure 9:**
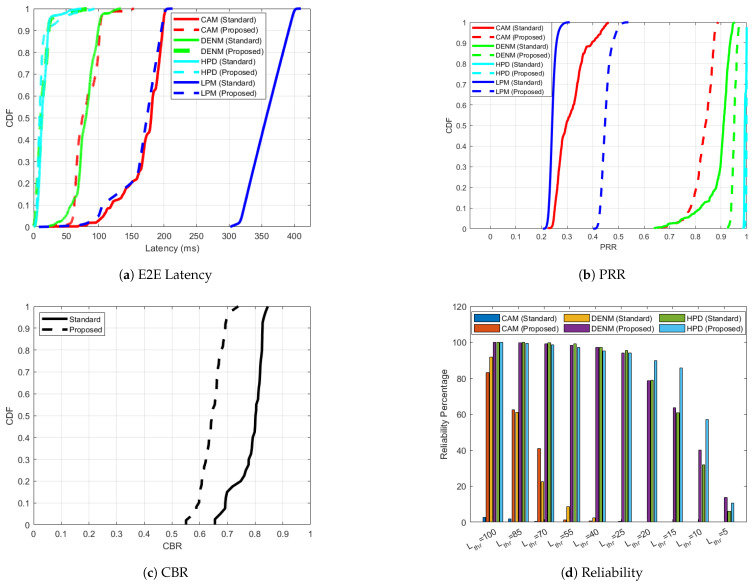
C-V2X PC5 performance improvement for the ‘Proposed’ versus ’Standard’ cases in scenario 4 with respect to one-way end-to-end latency, PRR, CBR and reliability.

The simulation results for C-V2X PC5 under low-speed conditions, with a maximum speed limited to 35 km/h, are shown in Figure 6, Figure 7, Figure 8 and Figure 9 for the four scenarios analyzed. Figure illustrates the performance comparison between the ’Standard’ and ‘Proposed’ approaches for C-V2X PC5 scenario 1, where all nodes exclusively transmit and receive CAMs. In the standardized case, represented as ‘CAM (Standard)’ in Figure 6a, CAMs experience high latency despite achieving PRR between 91–98% (Figure 6b) and CBR between 0.1 and 0.31 (Figure 6c). This is replicated in Figure 6d, where the CAM reliability worsens with higher latency thresholds, making them unsafe for modern vehicular applications. In the absence of HPDs and DENMs, CAMs are assigned a smaller RRI value of 50 ms by the adaptive RRI algorithm. The impact on latency is shown as a dashed line curve ‘CAM (Proposed)’ in Figure 6a and this improvement is similarly replicated in the reliability plot in Figure 6d. A slight improvement is observed in CBR and PRR in Figure 6c and Figure 6b, respectively.

Figure 7 shows the performance of C-V2X PC5 scenario 2 where along with the CAMs, all the nodes in the simulation also send high-intensity LPMs. In Figure 7a, the ’Proposed’ case shows significantly improved latency performance for both CAM and LPM traffic compared to the ‘Standard’ case. ‘CAM (Proposed)’ maintains a latency below 100 ms for a majority of packets, while ’CAM (Standard)’ exhibits higher latencies, extending up to 200 ms. For LPM traffic, the latency in ‘LPM (Proposed)’ is consistently lower than ’LPM (Standard)’, with a sharp improvement evident from the CDF curve. The PRR performance in Figure 7b is noticeably higher in the adaptive case compared to the standardized for both CAM and LPM traffic. The 95 percentile of PRR is 0.91 for ‘CAM (Proposed)’ as compared to 0.49 for ‘CAM (Standard)’. ‘LPM (Proposed)’ similarly shows a significant improvement in PRR compared to ‘LPM (Standard)’, highlighting the effectiveness of adaptive mechanisms in mitigating packet loss. The adaptive mechanism effectively reduces channel congestion, as indicated by the left-shifted CDF curve for the ’Proposed’ case, compared to the ‘Standard’ case in Figure 7c. Although CAMs also experience higher latency values as compared to scenario one because of high intensity LPM traffic, notably, in Figure 7d, ‘CAM (Proposed)’ succeeds in delivering nearly 90% reliability for latency threshold of 100 ms, whereas ‘CAM (Standard)’ struggles to exceed 4% in this moderate congestion scenario, emphasizing the advantage of adaptive resource management.

Figure 8 shows the performance of ‘Standard’ and ‘Proposed’ approach for C-V2X PC5 scenario 3 where along with CAMs and LPMs, 20% of the nodes also send DENMs. In Figure 8a, the adaptive approach achieves a significant reduction in latency across all types of messages, that is, CAM, DENM and LPM, compared to the standardized case. ‘DENM (Proposed)’ displays exceptional latency performance, with nearly all packets delivered within 50 ms, highlighting the prioritization mechanism’s effectiveness for time-critical messages. ‘CAM (Proposed)’ also outperforms ‘CAM (Standard)’, maintaining a latency below 100 ms for most packets. ’LPM (Proposed)’ demonstrates consistent improvements over ’LPM (Standard)’, reflecting the adaptive approach’s versatility in handling different traffic types. The adaptive approach shows a notable increase in PRR Figure 8b for all message types compared to the standardized case. The 95 percentile value of PRR for ‘DENM (Proposed)’ improves to 0.99 compared to 0.84 for ’DENM (Standard)’. ‘CAM (Proposed)’ and ‘LPM (Proposed)’ also exhibit substantial PRR gains, confirming the adaptive method’s ability to reduce packet losses effectively. The 95 percentile value of CBR in Figure 8c is 0.44 for the ’Proposed’ case, compared to 0.66 for the ’Standard’ case, showing substantial improvement. In Figure 8d, ’DENM (Proposed)’ shows superior reliability, achieving nearly 95% reliability for stringent latency thresholds of 25 ms, while ’DENM (Standard)’ are less than 10% reliable even for 55 ms $L_{thr}$. In the presence of DENMs, ‘CAM (Proposed)’ still delivers significantly higher reliability compared to ’CAM (Standard)’, demonstrating the adaptive approach’s ability to maintain reliability for both time-sensitive and regular messages.

Similarly to scenario 4 for ITS-G5, the most constrained scenario for C-V2X PC5 is scenario 4. In this scenario, 60% of the nodes only send CAMs and LPMs. 20% of the nodes send DENMs, CAMs and LPMs. The remaining 20% of the nodes send HPDs, CAMs and LPMs. Figure 9 shows the performance of ‘Standard’ and ‘Proposed’ approach for C-V2X PC5 scenario 4. In Figure 9a, DENM and HPD traffic show outstanding latency performance in the adaptive case, with most packets delivered in 25 ms, while the standardized counterpart of DENM exhibits noticeably higher delays. ‘CAM (Proposed)’ outperforms ‘CAM (Standard)’, maintaining latencies below 100 ms for the majority of packets. The PRR for all message types in Figure 9b improves significantly under the adaptive ‘Proposed’ mechanism. ‘HPD (Proposed)’ achieves near-perfect PRR, highlighting its suitability for critical applications. ‘DENM (Proposed)’, ‘CAM (Proposed)’ and ‘LPM (Proposed)’ also display substantial PRR improvements over their ‘Standard’ counterparts. The 95 percentile value of CBR in Figure 9c is 0.84 for the ‘Standard’ case, which improves to 0.71 for the ‘Proposed’ case, in turn optimizing channel usage and successful delivery of C-ITS messages. The adaptive mechanism significantly improves reliability across all latency thresholds and message types, as shown in Figure 9d. ‘HPD (Proposed)’ achieves over 90% reliability for latency thresholds as low as 20 ms, demonstrating its effectiveness for time-sensitive data. ‘DENM (Proposed)’ and ‘CAM (Proposed)’ similarly outperform their ’Standard’ counterparts by considerable margins, ensuring timely delivery for critical applications.

To enhance clarity and facilitate comparison, the 95 percentile values of latency, PRR and CBR for both standardized and adaptive cases across all scenarios are consolidated in Table 11 for C-V2X PC5 at a low-speed of 35 km/h. Table 12 highlights the 95 percentile values for latency, PRR and CBR for all scenarios at a high speed of 120 km/h. A similar trend is evident in the C-V2X PC5 simulations at both low and high speeds, mirroring the behavior observed in ITS-G5.

## 8. Conclusions and Future Directions

This research highlights the pressing need for adaptive and intelligent mechanisms to address the limitations of standardized V2X communication protocols in dynamic high-density environments. By introducing novel algorithms for AC selection in ITS-G5 and RRI adjustment in C-V2X PC5, coupled with a priority-aware message rate adjustment mechanism, we have demonstrated a significant leap in managing multi-service, multi-priority vehicular communication. Extensive simulations using the NS3 framework validate the efficacy of our proposed solutions, showcasing notable improvements in one-way end-to-end latency, PRR and reliability compared to existing standardized approaches. A total of four scenarios, ranging from very lightly to extremely heavily congested, are tested to evaluate the performance validity. These findings pave the way for a robust and scalable framework, critical to realizing the full potential of ITS to enable safe and autonomous mobility.

In terms of future work, we intend to (i) design a load balancing mechanism for ITS-G5 and C-V2X PC5, where nodes can shift part of their load from one short-range V2X technology to the other; (ii) expand our intra-technology parameter optimization work by incorporating more relevant parameters and validating the strengths it adds to the short-range V2X communication. 

## Figures and Tables

**Table 1:** Mapping of ACs onto transmission parameters for ITS-G5 according to ETSI TS 102 636-4-2 V1.1.1 [3].

TC ID	AC	Channel	Maximum Transmit Power Level [dBm]	MCS	Intended Use
0	$AC_{VO}$	CCH	+33	6 Mbit/s	High-priority DENM
1	$AC_{VI}$	CCH	+23	6 Mbit/s	DENM
2	$AC_{BE}$	CCH	+23	6 Mbit/s	CAM
3	$AC_{BK}$	CCH	+23	6 Mbit/s	Multihop DENM, Other data traffic

**Table 2:** ETSI ITS-G5 Reactive DCC as specified in [4].

CBR	State	Packet Rate	T_off_
CBR ≤ 0.30	Relaxed	10 Hz	100 ms
0.30 < CBR ≤ 0.40	Active 1	5 Hz	200 ms
0.40 < CBR ≤ 0.50	Active 2	2.5 Hz	400 ms
0.50 < CBR ≤ 0.60	Active 3	2 Hz	500 ms
CBR > 0.60	Restrictive	1 Hz	1000 ms

**Table 3:** Variables used and their description.

Variable(s)	Description
$AC_{VO}$, $AC_{VI}$, $AC_{BE}$, $AC_{BK}$	Voice, Video, Best Effort and Background access categories
$AC_{CAM}$, $AC_{DENM}$, $AC_{HPD}$, $AC_{LPM}$	the selected access category for CAM, DENM, HPD and LPM packets
$TX_{CAM}$, $TX_{DENM}$, $TX_{HPD}$, $TX_{LPM}$	binary variables representing transmission status of CAM, DENM, HPD and LPM packets
$RX_{CAM}$, $RX_{DENM}$, $RX_{HPD}$, $RX_{LPM}$	binary variables representing reception status of CAM, DENM, HPD and LPM packets
$RRI_{CAM}$, $RRI_{DENM}$, $RRI_{HPD}$, $RRI_{LPM}$	Selected RRIs for CAM, DENM, HPD and LPM packets
$L_{ITS-G5_{X}}$	the one-way end-to-end latency via ITS-G5 for message type X
$L_{PC5_{X}}$	the one-way end-to-end latency via C-V2X PC5 for message type X
$t_{gen_{X}}^{ITS-G5}$, $t_{gen_{X}}^{PC5}$	generation timestamp for C-ITS message type X
$t_{rec_{X}}^{ITS-G5}$, $t_{rec_{X}}^{PC5}$	reception timestamp for C-ITS message type X
$N_{ITS-G5_{CAM}}$, $N_{ITS-G5_{DENM}}$, $N_{ITS-G5_{HPD}}$, $N_{ITS-G5_{LPM}}$	total number of CAM, DENM, HPD and LPM packets received via ITS-G5
$N_{PC5_{CAM}}$, $N_{PC5_{DENM}}$, $N_{PC5_{HPD}}$, $N_{PC5_{LPM}}$	total number of CAM, DENM, HPD and LPM packets received via C-V2X PC5
$T_{ITS-G5_{CAM}}$, $T_{ITS-G5_{DENM}}$, $T_{ITS-G5_{HPD}}$, $T_{ITS-G5_{LPM}}$	total number of CAM, DENM, HPD and LPM packets transmitted via ITS-G5
$T_{PC5_{CAM}}$, $T_{PC5_{DENM}}$, $T_{PC5_{HPD}}$, $T_{PC5_{LPM}}$	total number of CAM, DENM, HPD and LPM packets transmitted via C-V2X PC5
$PRR_{ITS-G5_{X}}$	Packet Reception Ratio via ITS-G5 for message type X
$PRR_{PC5_{X}}$	Packet Reception Ratio via C-V2X PC5 for message type X
$REL_{ITS-G5_{X}}$	C-ITS message reliability via ITS-G5 for message type X
$REL_{PC5_{X}}$	C-ITS message reliability via C-V2X PC5 for message type X
$L_{thr}$	one-way end-to-end latency threshold for reliability evaluation
$CBR_{thr1}$, $CBR_{thr2}$, $CBR_{thr3}$	CBR thresholds 1, 2 and 3
$AC_{CAM_{ST}}$, $AC_{DENM_{ST}}$, $AC_{HPD_{ST}}$, $AC_{LPM_{ST}}$	the standard Access Category for CAM, DENM, HPD and LPM packets
$S_{window}$	sliding time window duration in milliseconds
$F_{CAM}$, $F_{DENM}$, $F_{HPD}$, $F_{LPM}$	transmission frequency of CAM, DENM, HPD and LPM packets
$f_{min}$, $f_{max}$	minimum and maximum possible frequency
Speed	min-max normalized value of speed of the V2X node

**Table 4:** ETSI V2X Congestion-Control-Maximum CR limit per CBR range and packet priority [5].

CBR-Based PSSCH Transmission Parameter Configuration	PPPP1-PPPP2	PPPP3-PPPP5	PPPP6-PPPP8
CBR Measured	CR Limit	CR Limit	CR Limit
0 ≤ CBR measured ≤ 0.3	No limit	No limit	No limit
0.3 < CBR measured ≤ 0.65	No limit	0.03	0.02
0.65 < CBR measured≤ 0.8	0.02	0.006	0.004
0.8 < CBR measured ≤ 1	0.02	0.003	0.002

**Table 5:** Summary of related work regarding AC/RRI adaptation and priority-aware rate control for short-range V2X communication technologies. ‘✓’ denotes present and ‘×’ denotes absent.

Ref.	Technologies Considered		AC Allocation	RRI Allocation	Congestion Control	ITS Services Considered			
	PC5	ITS-G5/DSRC				CAM/BSM	DENM	HPD	LPM/CPM
[7]	×	ITS-G5	Static	×	priority-aware	✓	✓	×	✓
[8]	×	ITS-G5	Static	×	standardized	✓	×	×	✓
[9]	×	DSRC	Adaptive	×	standardized	✓	×	×	×
[10]	×	ITS-G5	Static	×	priority-aware	✓	✓	×	✓
[11]	×	EDCA	Adaptive	×	standardized	×	×	×	×
[12]	×	EDCA	CW, AIFSN	×	priority-aware (single AC)	×	×	×	×
[13]	×	DSRC	CW, AIFSN	×	×	×	×	×	×
[14]	×	ITS-G5	Static	×	standardized	✓	×	×	✓
[19]	mode 4	×	×	Static	rate, power	×	×	×	×
[24]	mode 4	×	×	Adaptive	standardized	✓	×	×	×
[25]	mode 4	×	×	Adaptive	standardized	✓	×	×	×
[26]	mode 4	×	×	×	standardized	✓	×	×	×
[27]	mode 4	×	×	Static	standardized	✓	×	×	×
[28]	mode 4	×	×	Static	standardized	✓	×	×	×
[32]	mode 3	×	×	Static	standardized	×	×	×	×
[33]	mode 4	×	×	Static	rate, power	✓	×	×	×
[34]	mode 4	×	×	Static	speed-based rate	✓	×	×	×
[35]	mode 4	×	×	Static	standardized	✓	×	×	×
This work	mode 4	ITS-G5	Adaptive	Adaptive	priority-aware	✓	✓	✓	✓

**Table 6:** Mapping between Traffic Classes (TCs) and PPPP according to ETSI EN 303 613 V1.1.1 (2020-01) [6].

TC	PPPP	Intended Use
0	2	High Priority DENMs
1	4	Normal DENMs
2	5	CAMs
3	6	Forwarded DENMs and other low priority messages

**Table 7:** Simulation parameters.

Parameter	Value
Vehicular scenario	
Road length	3000 m
No. of vehicles	10
No. of lanes	1
Vehicle speed (max)	35 km/h, 120 km/h
Vehicle mobility	SUMO
Application layer	
Packet size—CAM	126 bytes
Packet size—DENM/HPD	141 bytes
Packet size—LPM (ITS-G5)	1850 bytes
Packet size—LPM (C-V2X PC5)	123 bytes
Default transmission frequency—CAM/DENM/HPD	10 Hz
Default transmission frequency—LPM (ITS-G5)	200 Hz
Default transmission frequency—LPM (C-V2X PC5)	500 Hz
MAC and PHY layer—ITS-G5	
Carrier frequency	5.9 GHz
Channel bandwidth	10 MHz
RSSI threshold	−94 dBm
Tx Power	23 dBm
Propagation model	Log Distance
Data Rate	6 Mbps
Modulation Scheme	OFDM
QoS/NQoS WaveMacHelper	QoS (for EDCA)
CBR thresholds ($CBR_{thr1}$, $CBR_{thr2}$, $CBR_{thr3}$)	0.25, 0.50, 0.75
MAC and PHY layer—CV2X PC5	
Carrier frequency	5.9 GHz
Channel bandwidth	10 MHz
No. of subchannels	1
Subchannel size	50 Resource Blocks
Resource keep probability	0
RSSI threshold	−90 dBm
Tx Power	33 dBm
Propagation model	Log Distance
MCS	20
HARQ enabled	false
Subchannelization scheme	Adjacency-PSCCH-PSSCH
Noise Figure	9 dB
CBR thresholds ($CBR_{thr1}$, $CBR_{thr2}$, $CBR_{thr3}$)	0.25, 0.50, 0.75

**Table 8:** Summary of scenarios with the percentage of vehicles transmitting each message type. ‘✓’ denotes present and ‘×’ denotes absent.

	CAM	LPM	DENM	HPD
Scenario 1	✓(100%)	X	X	X
Scenario 2	✓(100%)	✓(100%)	X	X
Scenario 3	✓(100%)	✓(100%)	✓(20%)	X
Scenario 4	✓(100%)	✓(100%)	✓(20%)	✓(20%)

**Table 9:** Ninety-fifth percentile values for latency, PRR and CBR in the ITS-G5 simulation conducted under low-speed conditions (35 km/h).

KPI	Message Type	Scenario 1		Scenario 2		Scenario 3		Scenario 4	
		Standard	Proposed	Standard	Proposed	Standard	Proposed	Standard	Proposed
Latency (ms)	CAM	2.31	–	142.81	4.14	118.04	3.38	168.6	10.35
	LPM	–	–	499.54	234.12	500.4	360.01	499.36	283.62
	DENM	–	–	–	–	4.99	3.5	3.94	3.55
	HPD	–	–	–	–	–	–	4.12	3.31
PRR	CAM	0.99	–	0.87	0.99	0.81	0.96	0.77	0.87
	LPM	–	–	0.59	0.76	0.53	0.73	0.43	0.71
	DENM	–	–	–	-	0.96	0.98	0.81	0.98
	HPD	–	–	–	–	–	–	0.91	0.99
CBR	–	0.07	–	0.89	0.65	0.94	0.73	0.98	0.84

**Table 10:** Ninety-fifth percentile values for latency, PRR and CBR in the ITS-G5 simulation conducted under high-speed conditions (120 km/h).

KPI	Message Type	Scenario 1		Scenario 2		Scenario 3		Scenario 4	
		Standard	Proposed	Standard	Proposed	Standard	Proposed	Standard	Proposed
Latency (ms)	CAM	2.22	–	157.12	6.91	129.93	6.90	199.84	30.53
	LPM	–	–	499.64	227.18	500.1	353.26	499.82	287.79
	DENM	–	–	–	–	8.43	6.84	8.11	7.63
	HPD	–	–	–	–	–	–	7.59	7.52
PRR	CAM	0.79	–	0.74	0.89	0.71	0.86	0.67	0.86
	LPM	–	–	0.52	0.74	0.41	0.65	0.34	0.61
	DENM	–	–	–	-	0.84	0.92	0.73	0.87
	HPD	–	–	–	–	–	–	0.81	0.95
CBR	–	0.05	–	0.71	0.51	0.77	0.57	0.79	0.59

**Table 11:** Ninety-fifth percentile values for latency, PRR and CBR in the C-V2X PC5 simulation conducted under low-speed conditions (35 km/h).

KPI	Message Type	Scenario 1		Scenario 2		Scenario 3		Scenario 4	
		Standard	Proposed	Standard	Proposed	Standard	Proposed	Standard	Proposed
Latency (ms)	CAM	97.78	21.99	195.41	102.13	198.42	102.05	199.84	103.55
	LPM	–	–	399.32	199.4	398.82	199.01	394.35	198.22
	DENM	–	–	–	–	103.17	27.36	102.3	26.32
	HPD	–	–	–	–	–	–	39.65	24.82
PRR	CAM	0.98	0.99	0.49	0.91	0.45	0.88	0.43	0.88
	LPM	–	–	0.27	0.49	0.26	0.49	0.27	0.48
	DENM	–	–	–	-	0.84	0.99	0.94	0.97
	HPD	–	–	–	–	–	–	0.99	1
CBR	–	0.31	0.22	0.55	0.41	0.66	0.44	0.84	0.71

**Table 12:** Ninety-fifth percentile values for latency, PRR and CBR in the C-V2X PC5 simulation conducted under high-speed conditions (120 km/h).

KPI	Message Type	Scenario 1		Scenario 2		Scenario 3		Scenario 4	
		Standard	Proposed	Standard	Proposed	Standard	Proposed	Standard	Proposed
Latency (ms)	CAM	103.51	24.51	182.99	99.99	202.98	103.98	189.82	103.78
	LPM	–	–	401.41	192.42	401.42	193.15	403.81	199.15
	DENM	–	–	–	–	103.12	28.91	102.21	24.26
	HPD	–	–	–	–	–	–	36.22	32.76
PRR	CAM	0.86	0.96	0.47	0.77	0.41	0.74	0.34	0.61
	LPM	–	–	0.20	0.42	0.21	0.40	0.20	0.40
	DENM	–	–	–	–	0.81	0.84	0.66	0.73
	HPD	–	–	–	–	–	–	0.90	0.97
CBR	–	0.14	0.13	0.53	0.36	0.6	0.40	0.83	0.69

## Data Availability

The raw data supporting the conclusions of this article will be made available by the authors on request.

## Abbreviations

The following abbreviations are used in this manuscript:

ABR	Average Blocking Rate
AIFSN	Arbitration Inter-frame Space Number
BSM	Basic Safety Message
CA	Cooperative Awareness
CAGR	Compound Annual Growth Rate
CAM	Cooperative Awareness Message
CBR	Channel Busy Ratio
C-ITS	Cooperative Intelligent Transport Systems
DENM	Decentralized Environmental Notification Message
DSRC	Direct Short Range Communication
EDCA	Enhanced Distribution Channel Access
IPG	Inter-Packet Gap
IPT	Inter-Packet Time
ITS-G5	Intelligent Transportation Systems-G5
KPI	Key Performance Indicators
LPM	Low Priority Message
PDR	Packet Delivery Rate
PPPP	ProSe Per Packet Priority
V2I	Vehicle-to-Infrastructure
V2N	Vehicle-to-Network
V2P	Vehicle-to-Pedestrian
V2V	Vehicle-to-Vehicle
V2X	Vehicle-to-Everything
